# *mcr*‐Mediated Colistin Resistance in *Escherichia coli*: A One Health Review of Reported Prevalence Across Human, Animal, Food, and Environmental Sectors

**DOI:** 10.1002/puh2.70376

**Published:** 2026-09-15

**Authors:** Ferdous‐Ul‐Haque Joy, Tashfiya Zaman Mouree, Munny Das, Asma Kabir

**Affiliations:** ^1^ Department of Pharmacy United International University Dhaka Bangladesh; ^2^ Department of Pharmacy Atish Dipankar University of Science and Technology Dhaka Bangladesh

**Keywords:** antimicrobial resistance, colistin resistance, *Escherichia coli*, *mcr* genes, One Health

## Abstract

Antimicrobial resistance (AMR) is a growing global health problem that threatens the effective treatment of bacterial infections. *Escherichia coli* is widely used as an indicator organism for monitoring AMR because it circulates among humans, animals, food, and the environment. As resistance to many commonly used antibiotics has increased, colistin has been reintroduced as a last‐resort treatment for severe infections caused by multidrug‐resistant Gram‐negative bacteria. The discovery of plasmid‐mediated colistin resistance genes (*mcr*) has raised serious concern, as these genes can spread easily between bacteria. This narrative review synthesizes findings from 28 selected studies published up to 2025, covering more than 36,000 *E. coli* isolates collected from human, animal, food, and environmental sources across different regions of the world. *mcr*‐positive *E. coli* were most commonly reported in livestock and meat products, with moderate occurrences in environmental samples and generally lower prevalence in human clinical isolates, although direct comparisons are limited by substantial study heterogeneity. These findings suggest that food‐producing animals and contaminated environments represent important reservoirs of colistin resistance, whereas human exposure may occur through indirect pathways, although prevalence data alone cannot establish transmission directionality. Even with restrictions on colistin use in animal production and improved antimicrobial stewardship in many countries, *mcr* genes persist due to factors like environmental contamination and co‐selection with other resistance genes. This persistence is influenced by environmental contamination, the presence of other resistance genes on the same plasmids, and global trade of food products. The available evidence underscores that colistin resistance cannot be effectively addressed within a single sector. A coordinated One Health approach that integrates human health, animal production, and environmental management is essential to limit the further spread of *mcr*‐mediated colistin resistance and to preserve the clinical value of this critical antibiotic.

## Graphical abstract

This One Health review maps the global prevalence of *mcr*‐mediated colistin resistance in *Escherichia coli* across human, animal, food, and environmental sectors. By tracking datasets across diverse geographic regions, the study highlights critical resistance hotspots, emphasizing the urgent need for integrated cross‐sectoral surveillance to limit global dissemination.

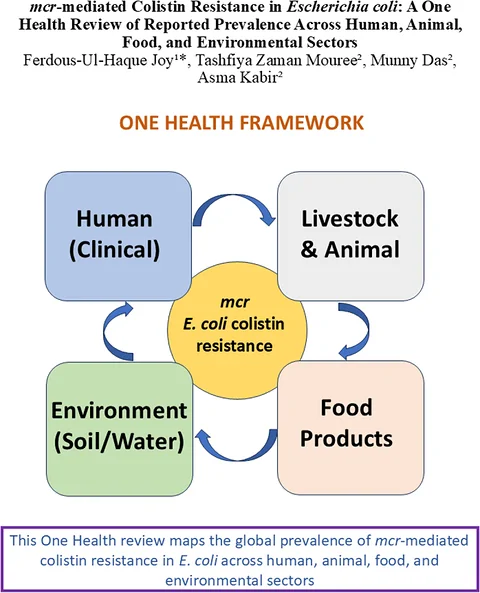

## 1 Introduction

Antibiotic discovery stands as one of the most significant medical advances of the 20th century. This single discovery dramatically reduced mortality from bacterial infections that were once fatal. However, bacteria have responded through natural selection and developed a range of mechanisms that allow them to survive against the antibiotics [1]. As a result, many antimicrobial drugs that were once highly effective have become less potent, contributing to the growing crisis of antimicrobial resistance (AMR) [2]. Current estimates suggest that AMR directly causes death around 700,000 annually, with projections indicating this could rise to 10 million deaths per year by 2050 if no effective action is taken [3].

*Escherichia coli*, a Gram‐negative bacterium predominantly found in the gastrointestinal tract of humans, is a common foodborne pathogen worldwide [4]. This bacterium enters the human body primarily through contaminated food sources such as undercooked meat, raw milk, contaminated fruits, and vegetables like lettuce and sprouts [5]. *E. coli* is responsible for a wide range of infections, including urinary tract infections, bloodstream infections, sepsis, and foodborne illnesses [5]. *E. coli* is considered a key Gram‐negative indicator of AMR because of its capacity to possess many resistance determinants and transmit resistance to potentially harmful or zoonotic bacteria [6]. Extended‐spectrum beta‐lactamase‐producing Enterobacteriaceae, including *E. coli*, are increasingly linked to significant public health issues due to restricted treatment alternatives. Carbapenems were previously regarded as the most effective antibiotics for treating severe infections caused by multidrug‐resistant (MDR) Enterobacteriaceae [7]. Nonetheless, the rising prevalence of carbapenem‐resistant Enterobacteriaceae (CRE) strains has led to a surge in the utilization of another class of antibiotic called polymyxins [8].

Polymyxin E, often known as colistin, is a polycationic antibiotic recognized for its effectiveness against Gram‐negative bacteria, especially Enterobacteriaceae [9]. Due to its significant nephrotoxic and neurotoxic effects, colistin is reserved as a last‐resort therapeutic option [10]. Despite these risks, this drug is regarded as an important treatment option when other antibiotics are ineffective [11]. Since 2016, the World Health Organization (WHO) has classified colistin as one of the critically important antibiotics [12]. Colistin is frequently employed to combat extensively drug‐resistant (XDR), pan‐drug‐resistant (PDR), and MDR bacterial infections that are typically unresponsive to other antibiotic classes [10]. Colistin has been used by veterinary professionals for many years to treat Gram‐negative bacterial infections [13]. Beyond its clinical use, colistin has been extensively employed as a growth promoter in livestock production, specifically in poultry, cattle, and pig farming [14]. The extensive misuse and overuse of colistin have contributed to the emergence of colistin‐resistant *E. coli*. In 2015, a study in China revealed the transferable colistin‐resistant *E. coli*, notably through the identification of *mcr‐* *1* gene from livestock and patients [15]. Within 4 years of its discovery, *mcr‐* *1*‐positive bacteria have been documented in animals, meat products, humans, and the environment in over 50 countries across five continents [16]. To date, 10 plasmid‐mediated colistin resistance genes (*mcr*‐*1* to *mcr*‐*10*) have been identified [17]. This global emergence of colistin resistance has become a serious concern considering the clinical importance of this antibiotic.

From a One Health perspective, we can't fully understand how *mcr*‐mediated resistance came about and spread without also looking at how it affected animals and the environment. A major cause of resistance selection is the very common use of colistin in veterinary medicine and food animal production, especially for disease prevention and promoting growth [18]. In the past, colistin has been administered to food‐producing animals at sub‐therapeutic doses, particularly in low‐ and middle‐income countries (LMICs). This has created selective pressure that makes it easier for *mcr*‐positive bacteria to spread and survive. Human exposure to these resistant organisms may occur through direct animal contact, contaminated food products, or environmental pathways.

The ecology of *mcr* genes is even more complicated by environmental compartments. When animal waste, untreated sewage, and pharmaceutical chemicals are released into the ground and water systems, they create new ecological niches for bacteria that are immune to drugs and mobile resistance elements [19]. Finding *mcr*‐positive *E. coli* in wastewater, farming soil, and surface water demonstrates that environmental compartments can serve as both reservoirs and transmission pathways for resistant bacteria [20, 21]. This interconnectedness makes it even more important to use surveillance methods that cover clinical, agricultural, and environmental areas all at once.

Even though more and more people are seeing the One Health sides of colistin resistance, reports still vary on how it spreads around the world and how much it affects different sectors. It is hard to get a clear picture of world trends because studies use so many different areas, sampling methods, and detection methods. Although some areas report markedly high prevalence in food sources and animal populations, others show low rates in clinical settings [22, 23]. This shows how different antibiotic stewardship, regulatory frameworks, and surveillance ability can be.

*E. coli* is widely recognized as a key sentinel organism for tracking AMR, and the continued detection of *mcr* genes across diverse settings highlights the need to view colistin resistance through a One Health lens. This review brings together evidence from selected studies that have examined the prevalence and distribution of *mcr*‐mediated colistin resistance in *E. coli* across human, animal, food, and environmental sectors. By outlining geographic trends, sector‐specific differences, and areas where these sectors intersect, the review provides an integrated overview of how colistin resistance is distributed and maintained. Unlike previous systematic reviews and meta‐analyses that have pooled prevalence estimates across bacterial species and geographic regions [16, 24], this review focuses exclusively on *E. coli* as a sentinel organism and explicitly examines sector‐specific patterns within the One Health framework. The value of this focused approach lies not in novel quantitative synthesis, but in tracing the epidemiological pattern of *mcr* persistence across interconnected compartments—identifying where resistance amplifies, where it persists after regulatory intervention, and where surveillance gaps remain. This is intended to inform targeted, sector‐specific policy response rather than to replace the quantitative work of prior meta‐analyses. This synthesis is intended to support clinicians, veterinarians, researchers, and policymakers in understanding the complex and interconnected nature of colistin resistance and the importance of coordinated action across sectors.

## 2 Methods

### 2.1 Review Design

This article is structured as a narrative review. Unlike formal systematic reviews, narrative reviews do not require exhaustive deduplication records or pooled statistical synthesis; however, this review applied structured selection criteria and a reproducible search strategy to improve transparency and reduce selection bias. This approach was chosen because the primary aim of the review was to map the distribution and sector‐specific patterns of *mcr*‐mediated colistin resistance in *E. coli* across One Health compartments rather than to derive a pooled prevalence estimate.

### 2.2 Literature Search

A structured literature search was conducted in PubMed/MEDLINE and Google Scholar using the following search terms and Boolean combinations: (*mcr* OR *mcr‐1* OR “mobile colistin resistance”) AND (*Escherichia coli* OR *E. coli*) AND (prevalence OR resistance OR surveillance OR One Health). Boolean operators and MeSH terms were applied in PubMed/MEDLINE; equivalent free‐text keyword strings were used in Google Scholar, which does not support controlled vocabulary indexing. Additional terms were combined with specific sectors: “livestock,” “human clinical,” “food safety,” “environmental,” “wastewater,” and “soil.” The search was restricted to publications available in English. Studies published from January 2015—the year of the landmark discovery of *mcr‐1—*through December 2025 were considered. Reference lists of included studies and relevant reviews were manually screened to identify additional eligible studies.

### 2.3 Study Selection

Studies were eligible for inclusion if they (i) reported the prevalence of any *mcr* gene variant (*mcr‐1* through *mcr‐10*) in *E. coli* isolates; (ii) specified the source sector (human clinical, animal/livestock, food, or environmental); (iii) provided sufficient data to calculate or verify a prevalence estimate (number of isolates tested and number of *mcr*‐positive, or a stated percentage); and (iv) were primary studies (i.e., not reviews, editorials, or case reports without prevalence data). Studies reporting *mcr* genes in bacterial species other than *E. coli* were excluded unless *E. coli* data could be extracted separately. Studies with unclear denominators, duplicate datasets without differentiated subgroup data, or without accessible full text were also excluded. Where multiple studies from the same research group used overlapping sample sets, only the study with the largest sample size or most complete data was retained. Secondary publications, including systematic reviews and meta‐analyses, were excluded as primary data sources for Table 1; Bastidas‐Caldes et al., a relevant systematic review and meta‐analysis of *mcr*‐mediated resistance, is cited for contextual framing only and was not used as a data source for this review. Screening was conducted by the lead author, with conflicts resolved in consultation with co‐authors. Final study selection was not performed using two independent reviewers, which represents a methodological limitation of this narrative review. The final literature search and screening process yielded 28 primary studies meeting all inclusion criteria, which form the evidence base synthesized in this review.

**TABLE 1:** Characteristics of included studies reporting *mcr*‐mediated colistin resistance in *Escherichia coli*.

Sl. no.	Country	Sector	Number of isolates (*N*)	*mcr‐*positive (*n*)	Prevalence (%)^a^	References
1	China	Raw meat, livestock, and human	2649	260	9.82	[15]
2	China	Livestock	1611	104	6.46	[25]
3	China	Livestock	130	75	57.69	[26]
4	China	Livestock	668	102	15.27	[27]
5	China	Livestock	1112	360	32.37	[28]
6	China	Livestock	2330	54	2.32	[29]
7	Bangladesh	Livestock	1200	305	25.42	[30]
8	Bangladesh	Livestock	104	14	13.46	[31]
9	Vietnam	Mixed	210	74	35.24	[32]
10	Pakistan	Human	120	6	5	[33]
11	India	Sewage water	253	5	1.98	[20]
12	Japan	Livestock	684	90	13.16	[34]
13	Japan	Livestock	9306	39	0.42	[35]
14	Arabian Peninsula	Human	75	4	5.33	[36]
15	Jordan	Livestock	360	143	39.72	[37]
16	Germany	Livestock	154	62	40.26	[38]
17 (a)	Germany^b^	Livestock	6158	709	10.42	[39]
17 (b)	The Netherlands^b^	Livestock	757	3	0.4	
17 (c)	Denmark^b^	Livestock	140	1	0.7	
17 (d)	Switzerland^b^	Livestock	129	0	0	
17 (e)	Belgium^b^	Livestock	113	20	17.7	
17 (f)	Poland^b^	Livestock	102	9	8.8	
17 (g)	Austria^b^	Livestock	73	0	0	
17 (h)	Spain^b^	Livestock	28	17	60.7	
17 (i)	Portugal^b^	Livestock	28	17	60.7	
17 (j)	Italy^b^	Livestock	42	25	59.5	
17 (k)	Hungary^b^	Livestock	12	4	33.33	
18	Multiple countries in Europe	Meat	128	33	25.78	[40]
19	France	Livestock	1701	49	2.88	[41]
20	Italy	Meat	147	2	1.36	[22]
21	Portugal	Human and companion animal	227	12	5.29	[42]
22	Argentina	Livestock	140	23	16.4	[43]
23	Brazil	Human	490	8	1.63	[23]
24	Nigeria	Clinical and nonclinical	35	3	8.57	[44]
25	Algeria	Soil, water, and manure	103	8	7.77	[21]
26	South Africa	Livestock	50	1	2	[45]
27	Tunisia	Human	676	4	0.59	[46]
28	Canada	Human	5571	2	0.04	[47]

^a^Prevalence calculation: Calculated as $(\frac{n}{N})\times 100$, indicating the percentage of isolates carrying any *mcr* gene variant.

^b^ *Multi‐country study metadata*: Rows 17 (a) through 17 (k) represent sub‐national data extracted from a single multi‐country surveillance study conducted by Ewers et al. [39]; data are broken down and reported separately per country. Rows 16 and 17 (a) represent two independent German investigations: Row 16 reports data specifically from Göpel et al. [38], focusing on pathogenic *E. coli* isolated from localized swine farms, whereas Row 17 (a) reports data from the broader longitudinal porcine surveillance study (2010–2020) by Ewers et al. *Methodological note*: Included datasets span various collection windows and geographic areas. Direct head‐to‐head comparisons across rows are mathematically constrained due to baseline heterogeneity in sampling metrics, molecular detection methodologies, target host animal species, and dynamic study timelines. Prevalence metrics should be interpreted as indicative of broad sector‐specific and regional resistome patterns rather than true population epidemiological snapshots.

### 2.4 Data Extraction

From each included study, the following data were extracted: country of study, study period, One Health sector (human clinical, animal, food, environment), *mcr* gene variant(s) detected, number of isolates tested, number of *mcr*‐positive isolates, and calculated prevalence (%). Where studies reported data across multiple sectors or *mcr* variants, data were extracted separately for each relevant subgroup. All extracted data were verified against the primary source by cross‐checking the stated prevalence against the raw numerator and denominator. Any discrepancies identified during this process are noted in Table 1 footnotes.

### 2.5 Data Synthesis

Due to substantial heterogeneity in study design, sample sources, geographic regions, detection methods, and study periods, formal meta‐analysis and quantitative pooling of prevalence estimates were not performed. This heterogeneity renders pooled estimates statistically and epidemiologically misleading. Instead, findings are synthesized narratively, with comparative patterns described across One Health sectors and geographic regions. Prevalence values should be interpreted as descriptive and sector‐specific rather than as generalizable population estimates. Where meaningful, quantitative patterns are described using ranges and sector‐level comparisons drawn directly from Table 1; no summary statistics were calculated across studies given that such calculations would obscure rather than illuminate the heterogeneity.

## 3 Colistin as the Last‐Resort Antibiotic

Colistin is an antibiotic belonging to cationic antimicrobial polypeptide named “polymyxins.” Polymyxins, including polymyxin B and polymyxin E (colistin), are reserved antibiotics primarily due to their toxicity. Colistin was first isolated in 1947 from *Paenibacillus polymyxa* subsp. *colistinus*, a Gram‐positive bacterium [10]. In 1959, US FDA approved the clinical use of colistin to treat Gram‐negative bacterial infections [48]. Since its discovery, its utilization has been restricted for several decades due to its adverse side effects in humans, primarily nephrotoxicity and neurotoxicity. It was replaced by safer classes of antibiotics. However, the global emergence of extended‐spectrum β‐lactamase (ESBL)‐producing and CRE revived the interest of colistin. It re‐emerged as a critical last‐resort drug when other antibiotics failed to work. WHO classified colistin as a “critically important antibiotic” for human since 2016 [49]. In hospital settings involving ventilator‐associated pneumonia, bloodstream infections and complicated urinary tract infections are the indications where colistin is the last choice of drug when other drugs are resistant.

Parallel to clinical use in humans, colistin has been used for many years by veterinary professionals in combating digestive infections commonly seen in pig and poultry farms caused by *E. coli*. Controversially, colistin sulfate is used as a growth promoter and as a metaphylaxis in livestock, particularly pig, poultry, and aquaculture in many countries [50]. The sub‐therapeutic, nonclinical application—prevalent in numerous low‐ and middle‐income nations prior to recent regulatory modifications—exerted significant selective pressure, promoting the creation and spread of resistance mechanisms, such as the plasmid‐mediated *mcr* genes.

The dual function of colistin in both human and veterinary medicine highlights its significance—and its susceptibility. Any diminution in effectiveness resulting from resistance would significantly limit available options for treating life‐threatening Gram‐negative infections. This reality has prompted demands for cautious utilization, limited veterinary interventions, and strengthened international surveillance, all within a One Health framework that acknowledges the interconnectedness of human, animal, and environmental health.

## 4 Emergence and Spread of *mcr*‐Mediated Colistin Resistance

The story of *mcr*‐mediated colistin resistance begins in 2015 when a team in China published the discovery of *mcr‐1*—the first plasmid‐mediated colistin resistance gene in *E. coli* isolates from pigs, retail meats, and humans (inpatients with infection) [15]. This was really a wake‐up call for everyone working on AMR around the world. Before that, it was believed that colistin resistance mostly came from the mutations in the bacterial chromosome that is relatively difficult to transfer between bacteria [51]. The fact that *mcr‐1* was carried on a mobile plasmid, so resistance could spread horizontally—not just vertically from parent to daughter cells—making it way more transmissible [52].

What made the finding even more concerning was how quickly *mcr‐1* had already disseminated. In just few years after the discovery of *mcr‐1*, it was reported in over 50 countries across all inhabited continents—in farm animals, companion animals, wildlife, food products, hospitalized patients, and environmental samples such as soil, sewage, and river water [33, 42, 43, 53, 54].

The primary driver behind this rapid spread was the extensive use of colistin in livestock. In many countries, especially across Asia, farmers routinely mixed colistin into animal feed as growth promoter and for disease prevention in poultry, swine, and aquaculture systems [55]. This sub‐therapeutic dosing created ideal conditions for selection and maintenance of resistant strains. The plasmids carrying *mcr‐1* (usually IncI2, IncX4, or IncHI2 types) proved to be highly conjugative, easily transferring between *E. coli* strains and even to other Enterobacteriaceae in animal guts [56, 57].

Soon after *mcr‐* *1*, new variants began to appear. *mcr‐* *2* was identified in 2016 in Belgian porcine and bovine isolates, *mcr‐* *3* in 2017 from Chinese swine, followed by *mcr‐* *4* and *mcr‐* *5* in European pigs and poultry [58, 59, 60]. Up until now, a total of 10 different *mcr* genes (*mcr‐* *1* to *mcr‐* *10*) have been identified [17]. Even though *mcr‐* *1* was found to be highly prevalent, *mcr‐* *3* and *mcr‐* *9* are increasingly reported in environmental and clinical settings [61, 62]. A particularly troubling feature of *mcr* genes is their frequent co‐location with other resistance determinants on the same plasmid. Many *mcr*‐positive *E. coli* isolates also carry ESBL genes (most commonly *bla*_CTX‐M_), carbapenemases (*bla*_NDM_ *or bla*_KPC_), and fluoroquinolone resistance markers (*qnr*) [63, 64].

Geographically, the highest prevalences have been documented in countries with historically heavy use of colistin in livestock. In Asia, Africa, and Latin America, the prevalence of colistin resistance in bacterial isolates has been alarmingly high [24]. The trade of food products, including animals, meat, and vegetables, may serve as a substantial catalyst for the global dissemination of *mcr* [65]. Moreover, the international commerce in animal meat and human travel facilitate the spread of *mcr* genes to nations with rigorous antibiotic regulations [66]. An underappreciated function of environmental compartments is that they may serve as mixing vessels where resistant strains from different sources encounter and exchange genetic elements, though the duration of *mcr* gene persistence in soil or water environments requires further direct measurement. This occurs in places like wastewater treatment plants, agricultural run‐off, and river systems [67]. Colistin bans in places, like China (2017) and Japan (2018), have brought down rates in some animal sectors [68, 69]. Veterinary medical products using colistin in conjunction with other oral antimicrobials had their marketing authorizations revoked by the European Commission in 2016 [70]. Colistin was outlawed in South Asian nations, including Bangladesh and India in 2019 and 2020, respectively [70].

Despite these initiatives, resistant genes still persist. From a One Health perspective, this persistence is logical: Once *mcr* genes get into the environment—through animal manure, wastewater, or contaminated soil, they can endure for years and transfer between compartments. Animal farms can still act as reservoirs even when colistin use drops, whereas wildlife, rivers, and irrigation water create pathways for resistance to come back into agriculture or reach people directly. On top of that, colistin is still used therapeutically in both animals and humans, which keeps the pressure on. And because *mcr* plasmids often carry other resistance genes, using different antibiotics can still help keep *mcr* around through co‐selection. Because of the close relationship between humans, animals, and the environment, simply outlawing colistin won't be sufficient to eradicate the resistance; instead, better stewardship and coordinated monitoring across all three sectors are required to truly break the cycle.

## 5 Prevalence and Distribution of *mcr*‐Mediated Colistin Resistance Across One Health Sectors

This section synthesizes findings from selected published studies reporting the prevalence of *mcr*‐mediated colistin resistance in *E. coli* across human, animal, food, and environmental sectors. This review considers all known *mcr* genes (*mcr*‐*1* to *mcr*‐*10*); no specific type was selected, and prevalence data include any reported *mcr*‐mediated colistin resistance in *E. coli*. Studies were chosen to cover diverse geographic regions, One Health compartments, and study designs, with emphasis on reports providing isolate‐level prevalence data and sufficient methodological detail. As this review is narrative rather than systematic or meta‐analytic, the included studies do not constitute an exhaustive compilation of all published reports, and some studies may have been excluded due to inaccessible data or methodological limitations. Consequently, the prevalence estimates presented here should be interpreted as indicative rather than definitive.

The fact that there are considerable differences in the prevalence of *mcr* genes in *E. coli* that have been isolated from different sources and nations is highlighted by the data that are shown in Table 1. The highest prevalence rates were found in livestock and food product samples, especially from Asia, the Middle East, and select European countries. In China, the prevalence of the *mcr* gene varied greatly, ranging from less than 3% to more than 50%. This varying prevalence highlights the significant heterogeneity that exists within the country, which is likely caused by changes in production methods, sample methodologies, and antimicrobial usage patterns. The high prevalence rate observed in the dataset from Tai'an, China (57.69%) [26], is attributed to targeted clinical sampling rather than a baseline surveillance layout. Isolates were derived exclusively from the livers of broiler chickens exhibiting overt clinical symptoms of avian colibacillosis (perihepatitis lesions). Furthermore, as noted by the primary investigators, non‐standardized veterinary antimicrobial administration in this specific region historically subjected these flocks to intense selective pressures, compounding high multidrug resistance profiles and co‐selection dynamics.

Asia was not the only region where high prevalence rates were observed. High detection rates were found in Vietnam, Germany, Spain, Portugal, and Italy. In certain instances, the frequency rates were found to be 40%–60%. On the basis of this situation, these findings suggest that *mcr*‐positive *E. coli* are reported across diverse geographic settings within animal production systems.

Contrarily, human clinical samples demonstrated a markedly lower prevalence of the *mcr*‐mediated resistance. There were low detection rates reported by researchers from Canada, Tunisia, Japan, and Brazil, despite the fact that they used high sample sizes. One possible contributing factor to these low prevalences may be the implementation of colistin restrictions. On the other hand, the presence of intermediate frequency levels in human isolates from Portugal, Pakistan, and the Arabian Peninsula suggests that human exposure is substantial and can be altered by a variety of factors, including animal reservoirs and dietary choices.

With respect to environmental sources, *mcr* genes were detected at generally low to moderate levels. Studies from Algeria and India reported modest detection rates in sewage and other ambient environmental samples. Although these levels were not among the highest observed, they remain noteworthy given the potential role of environmental compartments as persistent reservoirs and potential dissemination routes for *mcr* genes, though the duration of *mcr*‐positive *E. coli* survival in soil and water varies with environmental conditions and requires further direct measurement. In contrast, the relatively high occurrence reported in transnational European beef samples raises concern about foodborne exposure pathways and highlights the need for closer scrutiny of cross‐border transmission risks.

Taken together, the findings of this review illustrate how closely linked human, animal, food, and environmental compartments are within the One Health framework. Environmental sources appear to act as important interfaces where resistance determinants can persist and circulate, whereas the higher prevalence reported in animal and food‐related sources points to their role as key reservoirs, particularly in settings where antimicrobial use has been intensive. By comparison, the lower prevalence observed in human clinical isolates may reflect downstream exposure rather than primary amplification, although prevalence data alone cannot be used to infer transmission direction. Overall, the patterns summarized here suggest that resistance emerging or persisting in any one compartment has the potential to influence the others, underscoring the need to view these systems as dynamically interconnected rather than independent.

Reported prevalence varied widely across studies, reflecting substantial differences in sample size, sampling strategy, and study design. Smaller studies occasionally reported high prevalence values, which should be interpreted cautiously due to increased sampling variability. In contrast, larger surveillance‐based studies are likely to provide more stable prevalence estimates. Consequently, prevalence values across studies should be considered descriptive rather than directly comparable.

Substantial heterogeneity was observed within individual countries, with reported prevalence varying widely even within the same sector. These discrepancies likely reflect differences in animal species, production systems, regional antimicrobial use, sampling strategies, and study design rather than contradictory epidemiological trends.

The reviewed studies span periods both before and after the implementation of colistin restrictions in several countries. Due to heterogeneity in study design and sampling periods, direct quantitative comparison between pre‐ban and post‐ban prevalence was not undertaken. Nevertheless, several post‐ban studies report reduced prevalence of *mcr*‐positive *E. coli*, suggesting a potential impact of regulatory interventions on resistance dissemination. Several methodological factors limit cross‐study comparisons and must be considered when interpreting the data in Table 1. First, detection methods varied considerably: some studies employed phenotypic colistin susceptibility testing using broth microdilution (the gold standard per EUCAST/CLSI), whereas others used disk diffusion or agar dilution, which carry higher false‐positive and false‐negative rates for colistin [68]. Second, *mcr* gene confirmation methods ranged from multiplex PCR targeting specific variants to whole‐genome sequencing, which affects sensitivity for detecting novel or underrepresented variants. Third, studies with smaller sample sizes (*n* < 100) are prone to extreme prevalence estimates in either direction and should be interpreted with caution; the 57.69% prevalence in a Chinese livestock study of only 130 isolates illustrates this instability. Fourth, the sampling frame differed substantially. Some studies used convenience samples from farms, abattoirs, or clinics, whereas others employed systematic surveillance designs. These differences fundamentally affect the representativeness of findings. These methodological limitations do not invalidate the data but underscore the need to interpret Table 1 as a qualitative landscape of colistin resistance distribution rather than a basis for precise prevalence estimation.

## 6 One Health Transmission Pathways and Implications

The prevalence of *mcr*‐mediated colistin resistance in *E. coli* is a clear indication of the interrelationship between human, animal, and environmental health. This close relationship is supported by evidence of the 28 independent studies in these sectors. *mcr*‐positive isolates were most commonly found in livestock and meat samples, found in moderate amounts in the environmental sources, and less frequently in human samples across the regions. This trend indicates that epidemiological relationships exist in the cross‐section of sectors, where animals and the environment serve as one of the most crucial reservoirs and amplification sites of *mcr*‐positive *E. coli*. It is also worth mentioning that prevalence gradients are not sufficient to determine the transmission directionality, and evidence of cross‐sector transmission is better supported by molecular studies that show common plasmids or genetic relatedness between isolates.

Livestock are also seen to serve as large reservoirs in this interconnected system, especially in areas where colistin was used intensively in the past [71]. In China, Vietnam, India, Pakistan, and Bangladesh—where colistin has been widely used as a growth promoter and disease preventive in animals—the number of *mcr*‐positive isolates was significantly greater. Close interaction between animals and people, including farmers, slaughterhouse employees, and veterinarians, represents a biologically plausible route of exposure to *mcr*‐positive bacteria, though direct transmission chain evidence was not assessed in most included studies.

Another route of transmission is the food chain. In this route, meat that has been contaminated with *mcr*‐resistant *E. coli* may be a good method of transmission. A systematic review of *E. coli* prevalence in meat products confirmed widespread contamination across food categories, suggesting a plausible vehicle for *mcr*‐positive strains, though that review did not specifically characterize *mcr* carriage [72]. Individuals who are in contact with unprocessed meat or whose food is not cooked sufficiently are at a greater risk, and cross‐contamination in food preparation may also contribute to the rapid dissemination of resistance of bacteria [73]. This food source of entry is a potential clue to the presence of *mcr* genes in human isolates. The important yet frequently neglected environmental sources are the important source and route of resistance. Wastes, agricultural runoffs, and manure‐contaminated soil give good conditions to the survival of *mcr*‐positive *E. coli* and encourage horizontal transfer of plasmids among bacterial species [74]. In the table, sewage samples of India had a detection level of 1.98%, and soil and manure samples of Algeria had a detection level of 7.77%. The polluted rivers and irrigation water may bring resistant strains to the crops or directly expose human populations in the form of drinking, irrigation, or domestic use [75]. The resistance can be developed in wildlife by several ways of exposure and then spread to more geographical regions [76]. Despite colistin bans in many countries, the continued presence of *mcr* genes highlights the persistent interconnectedness of these transmission pathways.

Animal‐related incidence in Japan greatly decreased following the prohibition in 2018, but low levels remain. The same has been shown in Europe, where the general prevalence is small, yet still some lapses are being detected in food products and environmental samples. After being introduced into environmental reservoirs, *mcr* genes can be able to endure over a long period and might re‐enter the animal populations in a variety of ways, thus continuing to enable cross‐sector transmission [77]. In addition, ongoing therapeutic use of colistin and co‐selection by other antibiotics—often carried on the same plasmids—can maintain *mcr* genes even after direct colistin pressure is reduced.

Collectively, such findings highlight the fact that interventions, which are confined to just one sector, may not be effective. Even though this reduction in the prevalence of colistin in animal feed would be achieved through restriction of its use, the resistance can still exist or reoccur unless environmental contamination, wastewater management, and food safety are simultaneously considered. Notably, the settings with high prevalence rates are widespread in LMICs, and intensive farming activities are accompanied by poor surveillance capabilities and a lack of substitutes to colistin. Although the prevalence has decreased in Europe and North America due to stricter regulations, global trade and human mobility do not leave any country out of the spread of resistance.

Molecular and genomic evidence provides stronger mechanistic support for cross‐sector transmission than epidemiological prevalence patterns alone. Studies cited in this review and the broader literature identified clonal relationships between animal and human *mcr*‐positive *E. coli* isolates using whole‐genome sequencing. The Portugal study [42]—a notable example—demonstrated identical *mcr*‐*1*‐carrying plasmids shared between *E. coli* from cohabiting dogs and their owners, confirmed through long‐read sequencing and plasmid analysis. Similarly, the genomic characterization of predominant *mcr*‐*1*, *mcr*‐*4*, and *mcr*‐*5* *E. coli* in Spanish swine [56] identified IncI2 and IncX4 plasmid backbones previously reported in both animal and human clinical isolates, suggesting a shared mobile genetic element pool rather than independent emergence. However, most studies included here relied on prevalence data without molecular epidemiological confirmation; therefore, the inference of cross‐sector transmission pathways remains probabilistic rather than established for the majority of included studies.

Other than colistin resistance, most *mcr*‐positive isolates possess other resistance determinants, for example, ESBLs, carbapenemases, and fluoroquinolone resistance genes, which further lead to the development of MDR organisms. The decreased efficacy of colistin would significantly limit the available treatment of severe Gram‐negative infections, especially in healthcare institutions that are already strained by carbapenem‐resistant Enterobacterales [10].

The clinical implications of *mcr*‐mediated colistin resistance are most acutely felt in settings where colistin represents the last effective therapeutic option. Co‐carriage of *mcr* genes with carbapenemase‐encoding genes—particularly *bla*_NDM_ and *bla*_KPC_—creates isolates against which virtually no licensed systemic antibiotics retain reliable activity. Although the studies included in this review do not uniformly report co‐resistance profiles, the documented frequency of co‐carriage with ESBL genes (particularly *bla*_CTX‐M_) and fluoroquinolone resistance markers underscores that the clinical burden of *mcr*‐positive *E. coli* extends beyond colistin resistance alone [78]. Even in populations where *mcr* prevalence in clinical isolates appears low (e.g., <5% in Canada, Tunisia, and South Africa), the importation of resistant strains through travel, food trade, or animal movement represents an ongoing background risk for clinical exposure.

Overall, the cross‐sector patterns observed across these studies emphasize the urgent need for a coordinated One Health strategy that integrates human healthcare, animal production, and environmental management to control *mcr*‐mediated resistance.

## 7 Interventions and Future Directions

Addressing *mcr*‐mediated colistin resistance in *E. coli* requires moving beyond problem identification toward sustained, multisectoral solutions. Over the past decade, a variety of interventions have been applied with both success and failure, and the collective response is that a complete One Health approach is required, which combines human healthcare, animal farming, and environmental conservation.

One of the most impactful measures has been the removal of colistin as a growth promoter in animal feed. Following a major regulatory shift in the European Union to restrict veterinary colistin use in 2016 [70], China implemented an agricultural ban in 2017, followed by Japan, which banned colistin as a veterinary growth promoter in 2018, whereas therapeutic veterinary use continued. In several settings, this intervention produced measurable benefits. In Japan, the prevalence of *mcr*‐positive *E. coli* in swine declined from 13.16% to 0.42% between 2016 and 2021, likely reflecting reduced selective pressure in intensive farming systems. Similarly, lower prevalence in European livestock, such as in France and Germany, suggests that regulatory action combined with improved antimicrobial stewardship can curb resistance spread.

However, the available data also demonstrate that regulatory bans alone are insufficient. Despite the 2017 prohibition, some livestock studies from China continue to report high prevalence, reaching high values in smaller post‐ban samples. The findings are likely to be caused by persistent therapeutic use, partial compliance, and uneven enforcement. This condition is still observed to be high in nations like Bangladesh and Vietnam where intensive production of poultry and swine is accompanied with the lack of access to inexpensive substitutes. Similar cases can be traced in Jordan and Argentina that show the impact of economic and structural limitations. These findings underscore the fact that bans can be most effective with education of the farmers, availability of alternatives (e.g., probiotics, vaccines), and economic incentives.

Human healthcare practices have been found to be especially successful in antimicrobial stewardship programs. Guidelines in the international community recommend the use of colistin as a last‐resort antibiotic, and such practice seems to have led to extremely low prevalence in places like Canada and Tunisia. The use of targeted therapy instead of routine metaphylaxis has also been promoted by veterinary stewardship programs such as the EU prudent use campaigns. However, the stewardship practices are not useful in ensuring environmental persistence as *mcr* genes are still characterized in sewage and manure, which is not under the clinical prescribing control.

Sector bridging approaches are the most promising. Combined human, animal, and environmental surveillance systems also allow earlier identification of spillover and hotspots before they arise. Portugal's combined human–animal study illustrates how such integration can reveal potential household or occupational transmission routes. Surveillance of the environment, especially in wastewater treatment plants, has also brought to the fore the issue of urban transmission of *mcr* genes [79, 80, 81], which has led to the demand to treat effluents better. “Farm‐to‐fork” strategies—incorporating farm biosecurity, food safety monitoring, and public education—represent another step toward comprehensive control.

There are also growing efforts to eliminate the use of colistin. Other alternatives used in veterinary practice, like probiotics and bacteriophage therapy, have demonstrated promising outcomes in the reduction of *E. coli* infections, without the use of antibiotics [82]. Newer combination therapies and polymyxin analogs are being investigated in the field of human medicine to maintain therapeutic effects [83]. Interventions, like hi‐tech wastewater treatment technologies, have shown a possibility of curbing the spread of plasmid‐mediated resistance.

In spite of these developments, there are still major problems. Economic strains in most LMICs do not support alternative uptake, the enforcement of regulations is uneven, and the cross‐border trade can help in the re‐introduction of resistant strains. Co‐resistance also contributes to making the control work more difficult, as the administration of irrelevant antibiotics may keep *mcr*‐bearing plasmids even in the absence of exposure to colistin.

In the future, longitudinal surveillance of underrepresented areas, increased application of whole‐genome sequencing to trace plasmid spreading, and creation of global standards balancing agricultural demands and health protection of populations should be considered as a priority. Finally, the existing interventions have potential, but their effectiveness will be determined by the level of their integration at the intersection of all One Health sectors. Effective policies will be useful to learn and overcome the enduring gaps to retain colistin as the final line of defense against MDR Gram‐negative diseases.

## 8 Limitations of the Study

Formal prospective tracking of screening records at each stage of the literature search, including total records identified, duplicates removed, records excluded after title and abstract screening, and full texts assessed prior to final inclusion, was not maintained during the conduct of this review. Consequently, a PRISMA‐compliant flow diagram cannot be retrospectively reconstructed. This represents a transparency limitation inherent to the narrative review design and distinguishes this work from systematic reviews that mandate such documentation. The 28 included studies reflect the authors’ structured application of the inclusion and exclusion criteria defined in Section 2.3; however, the absence of a formal screening log means that the reproducibility of study selection cannot be fully verified by independent readers.

## 9 Conclusion

The widespread transmission of the colistin resistance mediated by the *mcr* in *E. coli* is a critical and long‐term health problem of the population. This plasmid‐borne gene first reported in China in porcine and human populations has spread within animal reservoirs, food products, aquatic food chains, and terrestrial substrates and, although with rare occurrence, in human clinical infections within 10 years. Evidence from 28 studies indicates that the highest prevalence is consistently observed in livestock and meat, particularly in regions with extensive colistin use in agriculture, whereas human hospital isolates generally remain below 10% in the studies reviewed here. The persistence and horizontal transfer of the genes encoding the resistance to colistin may occur with the help of the reservoirs of the environment, such as wastewater, manure, and riverine systems, so that the resistance may persist even after the decrease in agrarian use of colistin.

The regulatory bans on colistin as a growth promoter have proven to bring quantifiable improvement in the jurisdictions like China, the European Union, and Japan. However, this has still not been overcome due to the ongoing therapeutic use, co‐selection of concomitant antimicrobials, partial enforcement, and cross‐border contamination of the environment together with global trade systems that have enabled the spread of the resistant strains across the borders. Colistin is an important last‐line antibiotic, which is essential, and the co‐occurring prevalence of *mcr*, along with ESBL and other mobile resistance determinants compounds the risk to human health. In settings where carbapenem‐resistant Gram‐negative infections already limit treatment options, even low prevalence of clinical *mcr* carriage represents a disproportionate threat, as it forecloses the last available antibiotic option entirely.

The efficient alleviation of this problem will require a concerted One Health strategy that will combine human health, veterinary medicine, agriculture, and environmental management. Among the measures that will be important are the deployment of combined surveillance systems to interconnect clinical, agricultural, and environmental data; investing in sustainable options to livestock production like vaccines and probiotics; improving wastewater and manure management practices; and giving international assistance that will help LMICs to implement the interventions. Without such multisectoral activities, the further transmission of the *mcr*‐mediated colistin resistance may undercut the effectiveness of one of the few remaining treatment options against the MDR Gram‐negative pathogens.

## Author Contributions

**Ferdous‐Ul‐Haque Joy:** conceptualized the study, conducted the literature search and data curation, and prepared the original draft of the manuscript. **Tashfiya Zaman Mouree:** contributed to the literature review and assisted in writing and reviewing the manuscript. **Munny Das:** critically revised the manuscript and provided additional editing and feedback. **Asma Kabir:** supervised the project, offered guidance throughout the review process, and critically evaluated the manuscript. All authors have read and approved the final version of the manuscript and take responsibility for its content.

## Funding

The authors have nothing to report.

## Ethics Statement

This study is a narrative review of previously published literature and did not involve human participants or animals. Therefore, ethical approval was not required.

## Conflicts of Interest

The authors declare no conflicts of interest.

## Data Availability

Data sharing is not applicable to this article as no new data were generated or analyzed.
